# Effect of transcranial direct current stimulation on the number of smoked cigarettes in tobacco smokers

**DOI:** 10.1371/journal.pone.0212312

**Published:** 2019-02-14

**Authors:** Faisal Alghamdi, Ahmed Alhussien, Meshal Alohali, Abdullah Alatawi, Tariq Almusned, Shirley Fecteau, Syed Shahid Habib, Shahid Bashir

**Affiliations:** 1 Faculty of Medicine, King Saud University, Riyadh Saudi Arabia; 2 Laboratory of Canada Research Chair in Cognitive Neuroscience, CERVO Brain Research Center, Medical School, Laval University, Quebec, Canada; 3 Neuroscience Center, King Fahad Specialist Hospital Dammam, Dammam, Saudi Arabia; 4 Berenson-Allen Center for Noninvasive Brain Stimulation, Harvard Medical School, Boston, MA, United States of America; University Medical Center Goettingen, GERMANY

## Abstract

**Background:**

Recent studies reported that transcranial direct current stimulation (tDCS) applied over the dorsolateral prefrontal cortex (DLPFC) reduced craving and cigarette smoking. We aimed to evaluate whether 3 sessions of tDCS over the DLPFC modulate cigarette smoking which is a critical factor in tobacco smokers.

**Methods:**

In a double-blinded, sham-controlled, parallel experimental study, 22 participants who wished to quit smoking received tDCS with the cathodal over the right DLPFC and anodal over the left DLPFC based on the 10–20 EEG international system (F4, F3) at an intensity of 1.5 mA for 20 minutes during three consecutive days. For sham stimulation, the electrodes placement was the same as for the active stimulation.

**Results:**

For the short time interval (8 days after the end of the tDCS regimen), the number of smoked cigarettes was reduced similarly in the active and sham groups (*p* < 0.001). Also, at the long time-interval (4 months after the end of the tDCS regimen) as compared to pre-tDCS, there was no significant difference in the number of smoked cigarettes in the active (*p* = 0.806) or the sham (*p* = 0.573) groups. Overall, there were no statistically significant differences between the active and sham tDCS groups on cigarette smoking.

**Conclusion:**

These findings suggested that 3 sessions of tDCS over the right and left DLPFC may reduce number of smoked cigarettes for short-time period but might not be significantly more effective than sham to decrease cigarette smoking.

## Introduction

Tobacco smoking is one of the leading causes of mortality and morbidity worldwide. Smoking affects many systems in the body and causes serious and life-threatening conditions, such as lung cancer, stomach cancer, oral cancer, cardiovascular diseases and osteoporosis [1–6]. According to the world health organization (WHO), tobacco smoking kills up to half of its users, with an estimated 7 million deaths per year [7]. Tobacco users who die prematurely deprive their families of income, raise the cost of healthcare and hinder economic development [7]. It is a very complex disease for which treatment is still a challenge. It has been estimated that, globally, smoking causes over 500 billion US dollars in economic damage each year [8]. In Saudi Arabia, there are about six million smokers between the ages of 17 and 40 [9].

To quit smoking and maintain smoking cessation is not easy as tobacco dependence is a cluster of behavioral, cognitive and physiological phenomena. The majority of tobacco smokers who attempt to quit smoking fail to achieve their goal. Of these smokers, 51.1% have attempted to quit smoking. Of the smokers who attempted to quite, 88% could not last a year [10]. The high rate of relapse in smoking cessation is strongly induced by a craving for nicotine [11].

Many methods include cognitive-behavioral therapies, nicotine-replacement therapies, pharmaceutical treatments such as bupropion and varenicline [12] and combination of these techniques [13] to help people quit smoking have been introduced in the past year; however, despite the crucial importance for treatments to aid the cessation of smoking, favorable outcomes have not been significant in the long term [14]. Some of the options that have been explored are novel drugs to treat nicotine dependence, novel ways of using existing medicines and increasing use of technology to support behavioral changes [15, 16].

The dorsolateral prefrontal cortex (DLPFC) is part of the brain network associated with craving and smoking cue-reactivity [15, 16, 17]. Non-invasive brain stimulation methods like repetitive transcranial magnetic stimulation (rTMS), a technique that can modulate focal cortical activity showed significant reduction in smoking craving [18], food craving [19] and cocaine craving [20] by applying to DLPFC. The DLPFC is also involved in risk taking and decision making processes, processes that are impaired in smokers [21]. We decided to explore whether modulating cortical activity in this area could change reduce in number of smoked cigarettes using another noninvasive method of brain stimulation namely, transcranial direct current stimulation (tDCS). tDCS is a safe, non-invasive brain stimulation technique that functions by altering electrical cortical excitability [22]. tDCS has been used in many medical experimental trials for the treatment of several diseases [23, 24] and for the control of addiction [25, 26, 27] with an amplitude of (1.5–2 mA) being recommended for safety concerns [28]. More precisely, one session of tDCS or repeated sessions (5 or 10 session) of tDCS over the right and left DLPFC can reduce cue-induced craving [27, 29, 30]. However, negative findings on craving [31] and smoking [32] have also been reported. One hypothesis that can explain these mixed findings is the number of tDCS sessions and follow-up of smoking outcome measurements. We thus believe that there is a crucial need for studies investigating the effect of repeated sessions of tDCS on smoking [33] and to measure smoking with longer follow-up period.

In this study, we targeted the DLPFC using tDCS rather than rTMS for several reasons. First, in this region, tDCS methods has not been studied, second offers an advantage in that the scalp sensation associated with stimulation only lasts for a couple of seconds. Therefore, in a sham-controlled trial, subjects can be adequately blinded to the condition they are receiving [34]. Second, tDCS is a simple, safe and inexpensive technique and the device is highly portable.

We hypothesized that 3 sessions of tDCS applied over the right and left DLPFC will decrease cigarette smoking in subjects who wished to quit. Based on results from a meta-analysis showing that noninvasive brain stimulation may have greater effect on craving when targeting the right DLPFC [15], we choose to apply the anode over F4 and the cathode over F3. Thus the aim of this study was to test whether tDCS, applied over the DLPFC (cathodal right /anodal left), following a randomized, sham-controlled and double-blind study will modulate the number of cigarettes in tobacco smokers.

## Methods

### Study design

**Fig 1:**
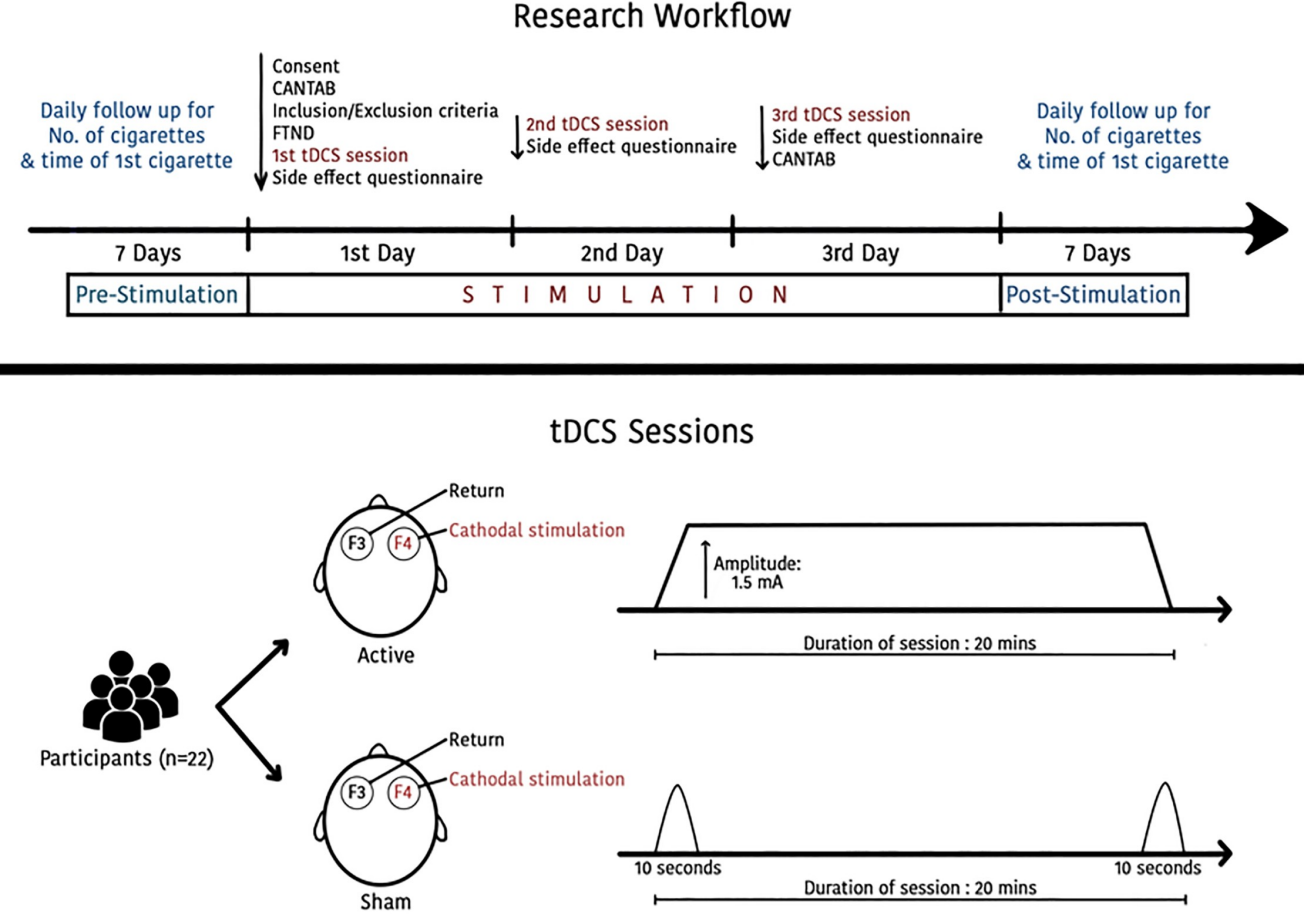
Flow sheet of experimental diagram.

This study was a randomized, parallel experimental double-blind, sham-controlled in which subjects received bilateral stimulation of DLPFC with tDCS: active cathodal right/anodal at F3 tDCS, and sham tDCS for three consecutive days. All session was performed at the same time of the day with same researchers at a 24-h inter-session interval time. Participants and the evaluating investigators (except the investigators that applied tDCS) were blinded to the treatment arm (**Fig 1**). Demographic data sheet were collected at baseline, which contains information about the purpose of the study and potential side effects from the device, and recording smoked cigarettes for the 7 days before the first day of stimulation. The following instruments of evaluation were used: 1-For baseline assessment: (i) smoking diary; (ii) Fagerstrom Test for Nicotine Dependence (FTND) questionnaire was filled to evaluate the addiction level for each subject [35] (iii) tDCS side-effect questionnaire (side effect checklist) (iv) Subjects were assessed again regarding their smoking diary (for seven days). 2-Subjects underwent tDCS treatment for 20 minutes for three days. 3-Post tDCS side-effect questionnaire after each tDCS session, smoking diary during the 3 days of tDCS and after the tDCS regimen for 8 days (collected for a total of 18 days), as well as at follow-up (4 months after the end of the tDCS regimen).

Fig 1 shows work flow plan of experimental design and areas of stimulation.

### Participants

A total of 22 male participants were recruited in the study. The safety of the participant was considered before applying any stimulation. Thus, each subject was screened through the use of a questionnaire before the first session [22]. Participants who were included were required to have been a smoker (at least 10 cigarettes per day) for one year or more and to be aged 18 years or older and wished to quit smoking. Subjects were recruited from local population with advertisement of study procedure. All subject did not have any psycho-educational help to quit smoking in past. The participants were divided into active (n = 12) and sham (n = 10) groups randomly by the operator. The mean age of the participants was 24.3 ± 5.03 years, and the mean number of cigarettes smoked per day was 20 ± 7.5 cigarettes (**Table 1**). As part of our inclusion/exclusion criteria, the participants should have no history or family history of neurological or psychological disorders, such as epilepsy, strokes or migraine. Additionally, participants were excluded if they had any history of neurosurgical/maxillofacial procedures, such as metal insertion or if they presented with any skin disorder at or near the stimulation locations (where the electrodes were placed), such as eczema, rashes, or other skin defects. Finally and as part of exclusion criteria, participants were instructed to declare during the study if they are using any other nicotine products such as water-pipe and chewed tobacco. This study was conducted in the Department of Physiology of the College of Medicine and King Khalid University Hospital (KKUH), Riyadh. All procedures were conducted according to the Declaration of Helsinki [36]. None of the participants have received any type of brain modulation before. This project was approved by the Institutional Review Board (IRB) at King Khalid University Hospital.

**Table 1:** Demographic characteristics of 22 subjects.

	Active (n = 12)	Sham (n = 10)	P value
**Age, mean (range) (SD)**	24.3 (19–29) (5.30)	24.4 (19–29)(5.03)	0.98
**Subjects who attempted to quit previously**	8	8	
**Cigarettes smoked per day, mean (SD)**	19.94 (7.15)	20.27 (8.96)	0.92
**Years of smoking, mean (SD)**	4.83 (4.19)	4.50 (4.47)	0.86
**FTND score, mean (SD)**	4.25 (2.00)	5.5 (2.01)	0.16

**Abbreviations:** SD = Standard deviation, FTND = Fagerstrom Test for Nicotine Dependence.

### Smoking diary

Participants were given a calendar to record the daily number of smoked cigarettes 1 week before the 1^st^ day of stimulation. The number of daily smoked cigarettes was collected for the seven days before stimulation which we considered to be their baseline (B1), the three days during stimulation (S1, S2, and S3) and the eight days after the last session of stimulation (P1-P8). Also the time between waking-up and smoking the first cigarette was recorded for the same period. Then, after four months from the last stimulation (P9), we collected the mean number of smoked cigarettes for 1 week to assess the long-term effect of tDCS.

### Fagerström's Nicotine Dependence Test (FNDT)

The level of nicotine dependence was assessed through the FTND questionnaire (S1 Table) that was given to each subject prior to his first stimulation [35].

### Adverse events assessment

Each subject was given an adverse event questionnaire that has been translated into Arabic after each one of the three stimulation sessions which had the following questions (tingling, itching sensation, burning sensation, neck pain, scalp pain, headache, fatigue, difficulties in concentration, nervousness, sudden mood change, change in visual perception, unpleasant sensation, visual sensation, nausea, drowsiness and weather the subject still feels the stimulation or not)

### Transcranial direct current stimulation (tDCS)

During the stimulation, participants remained seated in a comfortable chair. The StarStim 8 is a noninvasive wireless t-DCS/EEG neurostimulator (NE Neuroelectrics, Barcelona, Spain). It was used to deliver the direct current sequentially. The StarStim 8 neurostimulator includes a wireless neoprene cap, based on the International 10–20 system, which was placed on the participants’ head by aligning the central CZ electrode position with the vertex (the intersection of the nasion-inion and the inter-aural line mid-point). An 25-cm^2^ sponge electrode (sponstim-8) that is specific to the StarStimNE device (Pi electrodes, Neuroelectrics) was placed over the right DLPFC at F4 (cathodal) and F3 (return electrode; **Fig 1**). The electrodes were connected to a control box device, which was wirelessly connected to a computer and communicated with the NIC software (version 1.2, Neuroelectrics). During cathodal stimulation, direct current was delivered from a current-control circuit in the battery-driven stimulator within the control box device. The current was set at 1.5 mA intensity and applied for 20 minutes. For the sham stimulation, electrodes were placed in the same position and participants received a short ramp up for 10-second at the beginning of the session and another 10-seconds ramp up at end.

### Data analysis

Data were analyzed using SPSS (IBM Corp. Released 2012. IBM SPSS Statistics for Windows, Version 21.0. Armonk, NY: IBM Corp.).

A Shapiro-Wilk test score (p-value >0.05) and visual inspection for Histogram, Normal Q-Q Plot and boxplot showed normal distribution for number of cigarettes smoked for both active and sham groups, with skewness = 0.673 (SE = 0.616) and kurtosis = -0.177 (SE = 1.191) for active group, and skewness = 1.392 (SE = 0.661) and kurtosis = 3.651 (SE = 1.279) for sham group.

Numerical data was expressed as mean, median and standard deviation (SD). We used a repeated measures analysis of variance in which the dependent variable was the number of smoked cigarettes with Stimulation Group (active, sham) as between-factor and Time (B1, S1, S2, S3, P-P8 and P9) as within-factor and interaction Group versus Time.

The safety data were qualitative and the assumption of expected frequency being <20% was not violated for tingling, itching, burning, headache, or feeling the stimulation on the right side after taking off the electrodes. We used Pearson’s chi-square test for comparing the presence of these side effects before and after stimulation. As the expected frequency assumption was violated for fatigue, difficulty in concentration, acute mode change, change in visual perception, unpleasant sensation, unpleasant sensation in vision, nausea, drowsiness, and feeling the stimulation on the right side after taking off the electrodes, we used Fisher’s exact test for these side effects.

Two-tailed P values less than 0.05 were considered to be statistically significant. At the completion of the study, a total of 3 subjects were lost to attrition (2 dropouts after one session and one dropout after two sessions of tDCS). Data that could not be obtained were handled as missing at random.

## Results

The demographic data of the participants are shown in **Table 1.** There was no statistical difference between the active and sham groups in terms of the number of cigarettes smoked per day (*p =* 0.92), the number of years since smoking commenced (*p =* 0.86) and the FTND score (*p =* 0.16).

### Cigarette intake

**Fig 2:**
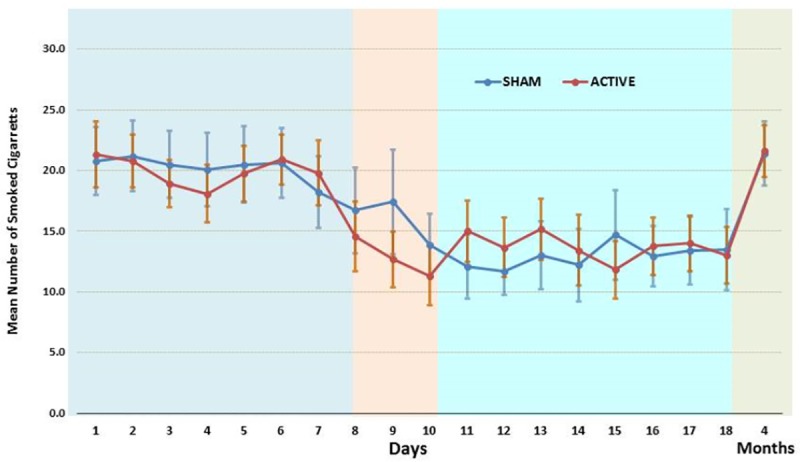
Daily number of smoked cigarettes before, during, after three days of stimulation, and after 4 months from stimulation.

The first objective was to test whether the number of smoked cigarettes was reduced when the participants received active stimulation compared to the participants that received sham stimulation (**Table 2**). The ANOVA revealed a significant main effect of time (F = 3.605; *p* = 0.038; η2 = 0.854), but no effect of Group (F < 0.01; *p* = 970; η2 < 0.001) and Time and Group interaction (F = 1.960; *p* = 0.024; η2 = 0.089). Interestingly, the number of smoked cigarettes was significantly reduced in both the active and sham groups (before and after stimulation, *p <* 0.000, **Fig 2**). Furthermore, during the interval of 15 days (7 days before stimulation and 8 days after the last stimulation session), the active group showed a significant change in number of smoked cigarettes after stimulation (*p <* 0.000). A change was also observed in the number of smoked cigarettes in the sham group, with a significant difference between the number of smoked cigarettes before and after stimulation (*p <* 0.000; **Table 2**). Moreover, there was no statistically significant difference in the percentage reduction (defined as the average number of smoked cigarettes post stimulation from day 11–18 divided by the average number of smoked cigarettes from day 1–7 multiplied by 100) between the active and sham groups during the interval of 6 days or 15 days (*p =* 0.76 and *p =* 0.649, respectively; **Table 3**). For the long-term-follow-up, we could not detect any significant changes between the baseline number of smoked cigarettes before the stimulation and after the four-months follow-up in the active or sham groups (*p =* 0.445 and *p* = 0.100 respectively; **Table 4)**.

**Table 2:** Number of smoked cigarettes pre-stimulation and 11 post-stimulation.

	Pre-stimulation (mean±SD)	Post-stimulation (mean)
**Active stimulation ^a^**	19.94±7.87	14.21±8.90***
**Sham stimulation ^a^**	20.27±8.86	14.40±9.60***
**Active stimulation ^b^**	19.94±7.87	13.49±8.40***
**Sham stimulation ^b^**	20.27±8.86	13.77±9.42***

15 days’ follow-up.

6 days’ follow-up

*** = <0.000

**Table 3:** Comparison of reduction percentage between active and sham.

	Active mean (SD)	Sham mean (SD)	*P* value
**Reduction % in 6 days**	34.28 (35.52)	30.11 (25.83)	0.647
**Reduction % in 15 days**	30.83 (32.50)	36.27 (22.20)	0.753

Abbreviations: SD = Standard deviation

**Table 4:** Comparing number of smoked cigarettes between mean number after 4 months of stimulation and before starting of stimulation / after stimulation.

	Pre-stimulation		Post-stimulation		Mean after 4 months (SD)
	Mean (SD)	*p* value	Mean (SD)	*p* value	
**All smokers (n = 18)**	20.1 (8.3)	0.57	13.65 (8.3)	0.01	21.5 (11)
**Active group (n = 10)**	19.9 (7.3)	0.45	13.5 (7)	0.01	21.6 (10.3)
**Sham group (n = 8)**	20.3 (10)	1.00	13.8 (10.1)	0.02	21.4 (12.7)

**Abbreviations:** SD = Standard deviation

### Side effects of tDCS

No subject dropped out of the study due to discomfort during the stimulation. The most frequent side effects were tingling sensation, itching, burning sensation at the electrodes sites and feeling of the stimulation on the left side after taking off the cap mostly in the active group **(Table 5).**

**Table 5:** Number of the occurrence of the side effects between active and sham groups.

	Total (n = 66)	Condition		*p*-value
		Active (n = 36)	Sham (n = 30)	
Tingling*	13	4	9	0.057
Itching*	21	14	7	0.208
Burning sensation*	13	8	5	0.599
Headache*	12	5	7	0.320
Fatigue**	7	3	4	0.695
Difficulty in concentration**	9	5	4	1.000
Sudden mode change**	10	4	6	0.335
Change in visual perception**	8	2	6	0.079
Unpleasant sensation**	5	4	1	0.372
Unpleasant sensation in vision**	11	9	2	1.000
Nausea **	4	2	2	1.000
Drowsiness **	6	3	3	1.000
Feeling the stimulation on right side **	10	8	2	0.105
Feeling the stimulation left side *	17	11	6	0.360

* Pearson Chi-Square test

** Fisher's Exact Test

## Discussion

To the best of our knowledge, this is the first sham-controlled study that evaluates the effects of 3 repeated tDCS sessions on smoking in adults with tobacco use disorders with a 4-month follow-up. We found that 3 tDCS sessions significantly reduced cigarette smoking in a short period following stimulation in the active as well as in the sham groups. The lack of significant effect between active and sham tDCS on cigarette smoking is contrary to our hypothesis and some previous studies measuring cigarette smoking [27, 29, 30]. These findings point out that we did not reach the optimal effect to minimize smoking-cigarette in smokers who wish to quit. This may be due to several factors, such as study design, stimulation parameters, and studied population.

Previous study showed nicotine abusers demonstrated area of the anterior cingulate; amygdala, insula, orbitofrontal and dorsolateral prefrontal cortex are associated with craving [14, 15]. As the effects of nicotine and other drugs might be connected to activity in mesolimbic dopamine pathways [37], this impact explains the contribution of the DLPFC (through the meso-fronto limbic connections).

Moreover, the use of a parallel design may have contributed to a stronger placebo effect as compared to previous studies using a crossover design [30]. Indeed, a recent meta-analysis investigated the impact of the study design on the placebo effect in noninvasive brain stimulation studies and revealed a significant effect of placebo in parallel studies but not in crossover studies [38]. The authors report [38] “that parallel design is a better approach than crossover design in testing the efficacy of the treatment.” Indeed, placebo effects in parallel design may represent some natural placebo responses, that would be more difficult to observe in crossover design as subjects can compare effects between the active and sham conditions (e.g., itching sensation related to tDCS). Subjects can still have such bias even when blinding assessment reports no significant statistical differences. This highlights the importance of conducting sham-controlled design, as well as blinding assessment with standardized questionnaire. Further, subject’s expectations that the treatment will help them to quit before delivering the intervention should be assessed to test whether this expectation level is linked to placebo responses. Placebo responses have been consistently reported in trials using various treatments for smoking cessation [39, 40]. Finally, inclusion of a no-treatment control in addition to a sham condition may provide insight on placebo responses in smokers that can involve several psychological factors including expectations, conditioning learning, memory, motivation somatic focus, reward, anxiety reduction, and meaning [39].Our study is limited by the fact that the sham group unfortunately had more drop outs. However, the link with the sham treatment is unlikely since participants dropped out before receiving any tDCS session. In addition, even though our study had the longest follow-up period among tDCS protocols for smoking cessation, one month may be too short to overcome acute placebo effects. Another explanation that may have contributed to the lack of significant findings for cigarette smoking is that the primary outcome is prone to a bias of underreporting. The inclusion of other outcomes such as latency to smoke or the total number of cigarettes smoked in one hour following the tDCS session would have been relevant, as done in Falcone et al. study [31].

Our comparison was performed over three-time intervals; a short interval of three days prior the stimulation and three days after stimulation, an intermediate interval of seven days before the stimulation and eight days after the last stimulation, and finally a time point four months after stimulation, which represents the long-time interval. Previous tDCS studies targeting stimulation of dorsolateral prefrontal cortex (DLPFC) revealed behavioral changes in healthy subjects [41, 42] and mood changes in patients with depression [43, 44].

We observed that there was a significant reduction in the number of smoked cigarettes during the short and intermediate time intervals; however, a reduction in the number of smoked cigarettes was not observed four-months after the stimulation period in either group.

The positive impact on smoking-reduction in the sham group, which appeared after the last session of stimulation, could be explained by the “placebo effect”. This effect depends mainly on two domains; a placebo-induced reaction and an interaction with the participant [45]. Moreover, the motivational effect, to which the participants were exposed prior and during sessions of stimulation, may have influenced the placebo group to produce a positive response to the placebo therapy as the placebo ritual was consistent with the person’s motives [46]. We cannot rule-out whether these psychological influences drove the sham group to record similar scores to the active group in our results. The present study aimed to promote smoking reduction by testing the role of a fundamental concept of decision-making proposed by Goldstein and Volkow using a noninvasive brain stimulation approach [47]. However, tDCS so far has shown variable effects on drug craving. A meta-analysis done by Jansen et al. (2013) showed that neuromodulation targeting the DLPFC could reduce craving levels for different substances [48]. From this analysis, five studies targeted the DLPFC for nicotine smoking. They showed a promising outcome with tDCS or rTMS on both the right and left sides. Fregni et al. showed that, in tobacco smokers, stimulation of both the left and the right DLPFC using anodal tDCS reduced the craving [18]. More recent studies have shown no effect when right and left anodal stimulation was performed over the DLPFC when it was used to test nicotine craving in smokers [32, 47, 49].

Moreover, since we recruited participants that showed high motivation in quitting smoking, another explanation of the lack of significant findings is the pure placebo response that could have mobilized the participants to pursue concomitant strategies to decrease smoking. However, to reduce this possibility, we specifically asked participants not to use nicotine-replacement strategies or medications for smoking-cessation during the protocol time course.

Boggio et al. demonstrated that bilateral tDCS decreased craving in alcoholics irrespective of the polarity (anodal left/cathodal right or cathodal left/anodal right) over the DLPFC [22]. Actually, neuroimaging studies showed both side of DLPFC involved for alcohol craving [50, 51]. Therefore, stimulating be cathodal inhibition of either right or left DLPFC ruptured the balance between the right and left DLPFC activity that might be normally necessary for craving states. Support for this notion of balanced bilateral activation of DLPFC during craving states has been shown in neuroimaging studies [15]. But Shahbabaie et al. found a state-dependent effect of anodal tDCS over the right DLPFC on methamphetamine craving [52]. Similar effects were obtained using repetitive stimulation of a comparable protocol but with the cathode over the left DLPFC and the anode over the right DLPFC, were a reduction in the probability of a relapse to the use of alcohol over a 6-month follow-up was observed [53]. With cathodal left and anodal right DLPFC stimulation, Fecteau et al. observed a decrease in the number of cigarettes smoked by tobacco smokers [30].

The side effects most commonly reported are mild headache, tingling, itching, burning sensation, and skin redness under the area of electrodes [2, 3, 11, 12, 19, 24]. Our results are in line with these findings. However, we also found a low frequency of these side effects. In our study, we did not find a significant difference in the amount of side effects reported between the active and sham stimulation groups for any of the interventions.

Although we showed that DC stimulation of the prefrontal cortex reduces smoking reeducation, further studies are needed to establish the use of tDCS as a viable clinical and therapeutic application. One potential advantage of developing tDCS as an alternative therapeutic strategy is the fact that the effects of tDCS are immediate. In addition, if tDCS does prove to have clinical value, it has additional advantage because it is safe and has a low incidence of only very mild adverse effects.

There are some limitations of this work. Although our sample size was larger than a lot of the previous studies that have assessed the effect of tDCS on craving, there is a need to re-evaluate these findings with a larger sample. Second, the motivational factors to quit smoking between the participants varied and this might have had an impact on the validity when we compared the active stimulation with the sham stimulation. Thus, it is hard to judge whether the reduction in the number of smoked cigarettes is because of the tDCS effect or because of stronger motivation to quit smoking in some of the participants compared to the others, which might be hard to accurately assess. Third, poor compliance during the follow-up was an issue and led to the exclusion of 5 subjects. Finally, we cannot determine, based on our findings, whether the effects of tDCS on reeducation of smoking cigarette were due to cathodal or anodal stimulation (or combination of both). Future studies using different electrode montages and sizes are critical to explore this matter further. Altogether, this evidence suggests that noninvasive brain stimulation might be an efficacious method to reduce different types of craving and thus further investigation with studies including larger sample sizes and also evaluating the clinical benefits of this treatment are warranted. Finally, the misconception in society about the device that as used in this study had its impact on the sample size that we obtained.

## Conclusions

In summary, we report that 3 sessions of tDCS applied over the right and left DLPFC reduced cigarette smoking in smokers. However, these beneficial cognitive and brain modulations were not sufficient to lead to a significantly higher decrease of cigarette smoking as compared to sham. tDCS was found to be safe in smoker adults. No significant difference was found in the frequency of side effects between the active and sham stimulation.

Despite the existing availability of tobacco addiction treatments, there are still smokers who are unable to quit using standard pharmacological and behavioral therapies. Although brain stimulation techniques suggested by other studies to be potentially useful treatment modality for tobacco addiction, the results from this work suggest that modulation of the right DLPFC through cathodal tDCS did not result in a significant difference between the active and sham groups. However, the desire to improve function can impede our understanding of the role of placebo effects. Placebo effects are well known in the context of medical interventions. In addition to the placebo effect, it may arise in any intervention when the desired outcome is known to the participant. Thus, improving cognitive abilities via tDCS is a powerful lure, raising important questions about the role of placebo effects in such studies. The question of whether reduction of smoking can be increased through stimulation has generated a lively scientific debate. Despite the limitations; this is the first study that has attempted to evaluate the effect of non-invasive brain stimulation on smoking cessation in the Middle Eastern region.

## Supporting information

S1 Table Fagerstrom Test for Nicotine Dependence (FTND) questionnaire to evaluate the addiction level for each subject. (DOCX)

## References

[1] Kõks G, Fischer K, Kõks S. Smoking-related general and cause-specific mortality in Estonia. BMC Public Health. 2018 12;18(1):34. 10.1186/s12889-017-4590-3 PMC5517793 28724413

[2] Li J, Yang F, Li X, Zhang M, Fu R, Yin X, et al Characteristics, survival, and risk factors of Chinese young lung cancer patients: the experience from two institutions. Oncotarget. 2015 7; 10.18632/oncotarget.19183 PMC5687685 29179515

[3] Ellison-Loschmann L, Sporle A, Corbin M, Cheng S, Harawira P, Gray M, et al Risk of stomach cancer in Aotearoa/New Zealand: A Māori population based case-control study. Behrens T, editor. PLoS One. 2017 7;12(7):e0181581 10.1371/journal.pone.0181581 28732086 PMC5521812

[4] Mahapatra S, Kamath R, Shetty B, Binu V. Risk of oral cancer associated with gutka and other tobacco products: A hospital-based case-control study. J Cancer Res Ther. 2015;11(1):199 10.4103/0973-1482.143332 25879362

[5] Lee W, Hwang S, Choi H, Kim H. The Association Between Smoking or Passive Smoking and Cardiovascular Diseases using a Bayesian Hierarchical Model: Based on the 2008–2013 Korea Community Health Survey. Epidemiol Health. 2017 6;e2017026 10.4178/epih.e2017026 28728350 PMC5723911

[6] Ayo-Yusuf O, Olutola B. Epidemiological association between osteoporosis and combined smoking and use of snuff among South African women. Niger J Clin Pract. 2014;17(2):174 10.4103/1119-3077.127542 24553027

[7] WHO | Tobacco WHO World Health Organization; 2017;

[8] Ekpu VU, Brown AK. The Economic Impact of Smoking and of Reducing Smoking Prevalence: Review of Evidence. Tob use insights. 2015;8:1–35. 10.4137/TUI.S15628 26242225 PMC4502793

[9] Isa MM, El-Sabbagh OI. Alert Addiction among Young Students in Taif City in Western Area of Saudi Arabia. Int J Sci Res Publ. 2014;4(1):2250–3153.

[10] Gallus S, Bosetti C, Chatenoud L, La Vecchia C, Saeedi M Al, Basulaiman M, et al Long live the Italians! Prev Med (Baltim). BioMed Central; 2015 1;70(1):76–7. 10.1016/j.ypmed.2014.11.015 25448842

[11] Baker TB, Brandon TH, Chassin L. Motivational influences on cigarette smoking. Annu Rev Psychol. 2004;55:463–91. 10.1146/annurev.psych.55.090902.142054 14744223

[12] Cahill K., Stevens S., Perera R. & Lancaster T. Pharmacological interventions for smoking cessation: an overview and network meta-analysis. Cochrane Database Syst. Rev. 5, CD009329 (2013). 10.1002/14651858.CD009329.pub2 PMC8406789 23728690

[13] Stead L. F., Koilpillai P., Fanshawe T. R. & Lancaster T. Combined pharmacotherapy and behavioural interventions for smoking cessation. Cochrane Database Syst. Rev. 3, CD008286 (2016). 10.1002/14651858.CD008286.pub3 27009521 PMC10042551

[14] Zwar NA, Mendelsohn CP, Richmond RL. Supporting smoking cessation. BMJ. 2014;348. 10.1136/bmj.f7535 24423971

[15] Olbrich H.M., Valerius G., Paris C., Hagenbuch F., Ebert D., Juengling F.D., 2006 Brain activation during craving for alcohol measured by positron emission tomography. Aust. N.Z. J. Psychiatr. 40, 171–178 10.1080/j.1440-1614.2006.01765.x 16476136

[16] Mcbride D., Barrett S.P., Kelly J.T., Aw A., Dagher A., 2006 Effects of expectancy and abstinence on the neural response to smoking cues in cigarette smokers: an fMRI study. Neuropsychopharmacology10.1038/sj.npp.1301075 16598192

[17] Wilson S.J., Sayette M.A., Fiez J.A., 2004 Prefrontal responses to drug cues: a neurocognitive analysis. Nat. Neurosci. 7, 211–214. 10.1038/nn1200 15001989 PMC2637355

[18] Eichhammer P., Johann M., Kharraz A., Binder H., Pittrow D., Wodarz N., Hajak G., 2003 High-frequency repetitive transcranial magnetic stimulation decreases cigarette smoking. J. Clin. Psychiatr. 64, 951–953 10.4088/jcp.v64n0815 12927012

[19] Uher R., Yoganathan D., Mogg A., Eranti S.V., Treasure J., Campbell I.C., Mcloughlin D.M., Schmidt U., 2005 Effect of left prefrontal repetitive transcranial magnetic stimulation on food craving. Biol. Psychiatr. 58, 840–842 10.1016/j.biopsych.2005.05.043 16084855

[20] Camprodon J.A., Martinez-Raga J., Alonso-Alonso M., Shih M.C., PascualLeone A., 2007 One session of high frequency repetitive transcranial magnetic stimulation (rTMS) to the right prefrontal cortex transiently reduces cocaine craving. Drug Alcohol Depend. 86, 91–94. 10.1016/j.drugalcdep.2006.06.002 16971058

[21] Engelmann J. M. et al Neural substrates of smoking cue reactivity: A meta-analysis of fMRI studies. NeuroImage 60, 252–262 (2012). 10.1016/j.neuroimage.2011.12.024 22206965 PMC3288122

[22] Bikson M, Datta A, Elwassif M. Establishing safety limits for transcranial direct current stimulation. Clin Neurophysiol. NIH Public Access; 2009 6;120(6):1033–4. 10.1016/j.clinph.2009.03.018 19394269 PMC2754807

[23] Meron D, Hedger N, Garner M, Baldwin DS. Transcranial direct current stimulation (tDCS) in the treatment of depression: Systematic review and meta-analysis of efficacy and tolerability. Neurosci Biobehav Rev. 2015 10;57:46–62. 10.1016/j.neubiorev.2015.07.012 26232699

[24] Brunoni AR, Shiozawa P, Truong D, Javitt DC, Elkis H, Fregni F, et al Understanding tDCS effects in schizophrenia: a systematic review of clinical data and an integrated computation modeling analysis. Expert Rev Med Devices. 2014 7;11(4):383–94. 10.1586/17434440.2014.911082 24754366

[25] Boggio PS, Sultani N, Fecteau S, Merabet L, Mecca T, Pascual-Leone A, et al Prefrontal cortex modulation using transcranial DC stimulation reduces alcohol craving: A double-blind, sham-controlled study. Drug Alcohol Depend. 2008;92(1–3):55–60. 10.1016/j.drugalcdep.2007.06.011 17640830

[26] Batista EK, Klauss J, Fregni F, Nitsche MA, Nakamura-Palacios EM. A randomized placebo-controlled trial of targeted prefrontal cortex modulation with bilateral tDCS in patients with crack-cocaine dependence. Int J Neuropsychopharmacol. 2015;18(12):1–11. 10.1093/ijnp/pyv066 PMC4675977 26065432

[27] Fregni F, Liguori P, Fecteau S, Nitsche MA, Pascual-Leone A, Boggio PS. Cortical stimulation of the prefrontal cortex with transcranial direct current stimulation reduces cue-provoked smoking craving: A randomized, sham-controlled study. J Clin Psychiatry. 2008;69(1):32–40. 18312035

[28] Zhao Haichao, et al "Modulation of brain activity with noninvasive transcranial direct current stimulation (tDCS): clinical applications and safety concerns." Frontiers in psychology 8 (2017): 685.‏ 28539894 10.3389/fpsyg.2017.00685 PMC5423956

[29] Boggio P. S. et al Cumulative priming effects of cortical stimulation on smoking cue-induced craving. Neurosci. Lett. 463, 82–86 (2009). 10.1016/j.neulet.2009.07.041 19619607

[30] Fecteau S. et al Modulation of smoking and decision-making behaviors with transcranial direct current stimulation in tobacco smokers: a preliminary study. Drug Alcohol Depend. 140, 78–84 (2014). 10.1016/j.drugalcdep.2014.03.036 24814566 PMC4242508

[31] Falcone M. et al Transcranial Direct Current Brain Stimulation Increases Ability to Resist Smoking. Brain Stimul. 9, 191–196 (2016). 10.1016/j.brs.2015.10.004 26572280 PMC4789149

[32] Xu J., Fregni F., Brody A. L. & Rahman A. S. Transcranial direct current stimulation reduces negative affect but not cigarette craving in overnight abstinent smokers. Front. Psychiatry 4, 112 (2013). 10.3389/fpsyt.2013.00112 24065930 PMC3778370

[33] Fraser PE, Rosen AC. Transcranial Direct Current Stimulation and Behavioral Models of Smoking Addiction. Front Psychiatry. 2012;3:79 10.3389/fpsyt.2012.00079 22969733 PMC3431716

[34] Gandiga P.C., Hummel F.C., Cohen L.G., 2006 Transcranial DC stimulation (tDCS): a tool for double-blind sham-controlled clinical studies in brain stimulation. Clin. Neurophysiol. 117, 845–850 10.1016/j.clinph.2005.12.003 16427357

[35] Fagerström K, Kunze M, Schoberberger R, Breslau N, Hughes J, Hurt R, et al Nicotine dependence. Cross cultural comparisons in population surveys and cessation samples. Tob Control. 1996;5:52–6. 8795860 10.1136/tc.5.1.52 PMC1759482

[36] Association WM. World medical association declaration of helsinki: Ethical principles for medical research involving human subjects. JAMA. 2013 11 27;310(20):2191–4. 10.1001/jama.2013.281053 24141714

[37] Berridge K.C., Robinson T.E., 1998 What is the role of dopamine in reward: hedonic impact, reward learning, or incentive salience? Brain Res. Brain Res. Rev. 28, 309–369. 9858756 10.1016/s0165-0173(98)00019-8

[38] Dollfus S., Lecardeur L., Morello R. & Etard O. Placebo Response in Repetitive Transcranial Magnetic Stimulation Trials of Treatment of Auditory Hallucinations in Schizophrenia: A Meta-Analysis. Schizophr. Bull. 42, 301–308 (2016). 10.1093/schbul/sbv076 26089351 PMC4753589

[39] Moore RA, Aubin HJ. Do Placebo Response Rates from Cessation Trials Inform on Strength of Addictions? Int. J. Environ. Res. Public Health 2012, 9, 192–211; 10.3390/ijerph9010192 22470287 PMC3315081

[40] Zellweger JP, Boelcskei PL, Carrozzi L, Sepper R, Sweet R, Hider AZ. Bupropion SR vs placebo for smoking cessation in health care professionals. Am J Health Behav. 2005 May-Jun;29(3):240–9 15899687 10.5993/ajhb.29.3.5

[41] Fregni F., Boggio P.S., Nitsche M., Bermpohl F., Antal A., Feredoes E., Marcolin M.A., Rigonatti S.P., Silva M.T., Paulus W., Pascual-Leone A., 2005 Anodal transcranial direct current stimulation of prefrontal cortex enhances working memory. Exp. Brain Res. 166, 23–30. 10.1007/s00221-005-2334-6 15999258

[42] Kincses T.Z., Antal A., Nitsche M.A., Bartfai O., Paulus W., 2004 Facilitation of probabilistic classification learning by transcranial direct current stimulation of the prefrontal cortex in the human. Neuropsychologia 42, 113–117. 14615081 10.1016/s0028-3932(03)00124-6

[43] Fregni F., Boggio P.S., Nitsche M.A., Marcolin M.A., Rigonatti S.P., PascualLeone A., 2006a Treatment of major depression with transcranial direct current stimulation. Bipolar Disord. 8, 203–204. 16542193 10.1111/j.1399-5618.2006.00291.x

[44] Fregni F., Boggio P.S., Nitsche M.A., Rigonatti S.P., Pascual-Leone A., 2006b Cognitive effects of repeated sessions of transcranial direct current stimulation in patients with depression. Depress Anxiety 23, 482–484. 16845648 10.1002/da.20201

[45] Miller FG, Rosenstein DL. The nature and power of the placebo effect. J Clin Epidemiol. 2006;59(4):331–5. 10.1016/j.jclinepi.2005.12.001 16549251

[46] Hyland ME. Motivation and placebos: do different mechanisms occur in different contexts? Philos Trans R Soc Lond B Biol Sci. 2011;366(1572):1828–37. 10.1098/rstb.2010.0391 21576140 PMC3130400

[47] Goldstein RZ, Volkow ND. Drug addiction and its underlying neurobiological basis: Neuroimaging evidence for the involvement of the frontal cortex. Vol. 159, American Journal of Psychiatry. 2002 p. 1642–52. 10.1176/appi.ajp.159.10.1642 12359667 PMC1201373

[48] Jansen JM, Daams JG, Koeter MW, Veltman DJ, van den Brink W, Goudriaan AE. Effects of non-invasive neurostimulation on craving: a meta-analysis. Neuroscience & Biobehavioral Reviews. 2013 12 1;37(10):2472–80. 23916527 10.1016/j.neubiorev.2013.07.009

[49] Pripfl J, Tomova L, Riecansky I, Lamm C. Transcranial magnetic stimulation of the left dorsolateral prefrontal cortex decreases cue-induced nicotine craving and EEG delta power. Brain Stimul. 2014;7(2):226–33. 10.1016/j.brs.2013.11.003 24468092

[50] Olbrich H.M., Valerius G., Paris C., Hagenbuch F., Ebert D., Juengling F.D., 2006 Brain activation during craving for alcohol measured by positron emission tomography. Aust. N.Z. J. Psychiatr. 40, 171–178 10.1080/j.1440-1614.2006.01765.x 16476136

[51] George M.S., Anton R.F., Bloomer C., Teneback C., Drobes D.J., Lorberbaum J.P., Nahas Z., Vincent D.J., 2001 Activation of prefrontal cortex and anterior thalamus in alcoholic subjects on exposure to alcohol-specific cues. Arch. Gen. Psychiatr. 58, 345–352 11296095 10.1001/archpsyc.58.4.345

[52] Shahbabaie A, Golesorkhi M, Zamanian B, Ebrahimpoor M, Keshvari F, Nejati V, et al State dependent effect of transcranial direct current stimulation (tDCS) on methamphetamine craving. Int J Neuropsychopharmacol. 2014 10 1;17(10):1591–8. 10.1017/S1461145714000686 24825251

[53] Klauss J, Penido Pinheiro LC, Silva Merlo BL, Correia Santos G de A, Fregni F, Nitsche MA, et al A randomized controlled trial of targeted prefrontal cortex modulation with tDCS in patients with alcohol dependence. Int J Neuropsychopharmacol. 2014 11 1;17(11):1793–803. 10.1017/S1461145714000984 25008145

