# Supplementary material for: Effect of transcranial direct current stimulation on the number of smoked cigarettes in tobacco smokers
Source: PLoS One. 2019 Feb 14;14(2):e0212312. doi: 10.1371/journal.pone.0212312 (PMC6375608; doi:10.1371/journal.pone.0212312)
Supplement: S1 Table — (DOCX) [file pone.0212312.s001.docx]

| **SRG** | condition |  |  |  |
| --- | --- | --- | --- | --- |
| **1** | ACTIVE | 3 | Moderately Dependent | 2 |
| **2** | ACTIVE | 3 | Moderately Dependent | 2 |
| **3** | ACTIVE | 7 | Highly Dependent | 3 |
| **4** | ACTIVE | 6 | Highly Dependent | 3 |
| **5** | SHAM | 6 | Highly Dependent | 3 |
| **6** | SHAM | 7 | Highly Dependent | 3 |
| **8** | ACTIVE | 4 | Moderately Dependent | 2 |
| **10** | ACTIVE | 1 | Low Dependence | 1 |
| **11** | SHAM | 3 | Moderately Dependent | 2 |
| **12** | SHAM | 5 | Highly Dependent | 3 |
| **13** | ACTIVE | 7 | Highly Dependent | 3 |
| **14** | SHAM | 8 | Highly Dependent | 3 |
| **16** | ACTIVE | 3 | Moderately Dependent | 2 |
| **17** | ACTIVE | 3 | Moderately Dependent | 2 |
| **18** | SHAM | 6 | Highly Dependent | 3 |
| **19** | SHAM | 4 | Moderately Dependent | 2 |
| **20** | ACTIVE | 7 | Highly Dependent | 3 |
| **23** | ACTIVE | 3 | Moderately Dependent | 1 |
| **25** | ACTIVE | 4 | Moderately Dependent | 1 |
| **26** | SHAM | 4 | Moderately Dependent | 2 |
| **27** | SHAM | 7 | Highly Dependent | 3 |
| **28** | SHAM | 3 | Moderately Dependent | 1 |

S1 Table: Fagerstrom Test for Nicotine Dependence (FTND) questionnaire was filled to evaluate the addiction level for each subject.
